# Transactions of the American Medical Association, Instituted 1847

**Published:** 1850-12

**Authors:** 


### BIBLIOGRAPHICAL NOTICES.

---

*Transactions of the American Medical Association, instituted* 1847. Vol. III. Philadelphia, 1850. pp. 499.

The third volume of "Transactions of the American Medical Association," although less voluminous than its predecessor of the last year, is in no way inferior to it in the interest and character of its contents.

The meeting of 1850 was indeed in some respects, more encouraging and more fruitful in its results than either of the preceding ones. The number of delegates appointed by the different medical Societies, amounted to between six and seven hundred, from twenty-seven States of the Union, and although the larger number of these were not present, yet the fact of their appointment, shows the deep and wide spread interest which this new movement has inspired throughout the medical profession of the United States. It is believed that no body of physicians of equal number and respectability, has ever been assembled at one time, in any part of the world, and although its proceedings may as yet be inferior in point of scientific importance to the doings of many smaller bodies in other countries, yet the power and influence which it is destined to exert upon medical science in America, if held together by the ties of fraternal feeling, and managed with discretion and wisdom, can scarcely be estimated.

Based upon the representative system, and throwing open its portals to all the regularly educated members of the profession, in good standing with their brethren at home, without distinction of talent, wealth or position, the Association may be regarded as a great democratic medical assembly, in which all the members stand upon the same platform, and act in the true republican spirit.

Here the Professor, whose teachings and writings have rendered him eminent at home and abroad, and whose position gives him a deserved influence over the minds, both of his pupils and his contemporaries, places himself on the same level with the more obscure and humble private physician, whose name and whose medical skill may not be known beyond his immediate circle.

It is true that one element exists in the organization of the Association not strictly republican, viz., that which confers upon medical colleges the right of appointing delegates, irrespective of the medical societies of states and counties. The disturbing influence of this arrangement, has already been the cause of conflict between opposing interests, which has tended in some degree to mar the harmonious action of the Association, and has even induced a fear in some minds that a radical change will have to be made in this respect, before its full benefits can be realized. This, however, is a question upon which there is a variety of views, which it would be premature to discuss at this time.

The volume of Transactions before us, is as usual, chiefly occupied with the reports of the standing committees of the Association upon the several subjects committed to them. These reports with some exceptions, appear to us highly creditable to the talents and industry of the committees who framed them. In one respect, several of them are superior to the reports upon the same subjects in former years, inasmuch as they are strictly American, and contain altogether the original observations of our own men. While this curtails their length, it imparts to them freshness and originality, and brings them within the design contemplated by the Association. Such reports must tend to elevate our national character, and if continued in the same spirit, and improved from year to year, by the additional materials which American Physicians are continually furnishing, we shall soon evince to the world, that there are laborers in the field of Science here, who, as original thinkers, discoverers, and men of genius, have not their superiors in the older nations of Europe.

The first Report on Medical Sciences covering about 50 pages of the Transactions, and embracing a review of the several departments of Anatomy, Physiology, General Pathology, and Therapeutics, with other branches of natural science, bearing directly on the condition and progress of medical knowledge in America, is particularly rich and interesting. It would be impossible in our narrow limits to do more than glance at its general features, without specifying particular points.

An article on the arrangement of the cancelli in human bones, exhibits a novel and ingenious view of the mechanical uses of the interior structure of bone, which manifests in a striking manner, that admirable adaptation of means to ends, which prevails throughout all the works of nature. Dr. Wyman, the author, a distinguished Anatomist of Boston, deserves great credit for his curious investigations into this subject,

Under the same head are detailed the results of Dr. Morton's investigations, into the "capacity of crania in different races of man," a new and interesting subject, which is just beginning to attract attention. Dr. John Neill, and Dr. Joseph Leidy, both of Philadelphia, and amongst the most acute and industrious laborers in the field of Anatomy and Physiology, have also added their portion to this department in several interesting communications. The article on "Parasitic Life" by the latter gentleman, is particularly valuable, as well as the observations on the parasites which infest the human teeth, by Dr. H. J. Bowditch, of Boston. The fact that the number of parasites which are found on the teeth, is in proportion to the degree of cleanliness observed; and that the various detergent tooth washes do not impair their vitality, while soap destroys them instantly, if it should be confirmed by future observations, would possess great practical value.

The department of Pathology in the report, treats of the propagation of epidemic and contagious diseases, in which the views recently propounded on this subject by Drs. J. K. Mitchell, J. Knight, and S. H. Dickson, are briefly detailed. The progress of cholera is traced, and the interesting report of the post-mortem appearances in this disease, presented to the College of Physicians of Philadelphia, by a committee appointed to investigate the subject, is briefly sketched.

Dr. Austin Flint's observations on Serous Effusion, within the arachnoid cavity, receive a due share of attention, and are well worthy of examination, as are the facts on Etherization in Insanity communicated by Dr. Luther V. Bell and Dr. Ray.

A curious case of spontaneous Hydrophobia, reported to the Philadelphia College of Physicians by Dr. Condie, finds a place in this connection, as amongst the medical curiosities of the year. Some interesting observations made in the same body by Drs. Condie and Riofrey, on the causes of Pulmonary Consumption, are worthy of note—as also the abstract of an instructive paper on Intestinal Auscultation, by Dr. C. Hooker of Yale College.

This department of the report closes with a section on the formation and composition of Urinary Calculi, in which is recorded the results of analysis of the calculi in the large collection of Transylvania University by Professor Peter, as originally published in the Western Lancet—and also of the collection in the Museum of the Boston Society for Medical Improvement. The connection of different urinary deposits with the geological character of the earth, is appropriately urged by the committee as an inquiry well worthy of the investigation of physicians in the different parts of our country.

Dr. G. Emerson of Philadelphia, a member of the committee, has communicated a valuable section on "Vital Statistics," pointing out some of the causes of mortality in children of the two sexes. It appears from his inquiries that the preponderance of male over female births, which is known to exist, is soon lost by reason of the greater mortality of males, "so that the numbers of the two sexes living at the age of ten years, are very nearly equal, and at the fifteenth year, the living females outnumber the males, about as much as the males did the females at birth." Dr. E. supposes that the excess of mortality amongst male children, arises from the greater prevalence amongst them of the inflammatory affections of the organs of respiration, circulation and nutrition, but more especially inflammation of the brain and its appendages,—while "the principal diseases of which females die, are hooping cough, small pox, scarlet fever, measles, thrush and consumption." The diseases of the males being allied to the sthenic, and of the females to the asthentic class.

Dr. E. also presents some very curious enquiries upon the "Influences operating to change the number of births, and also the proportion of the sexes at birth." The seasons, the quantity and quality of the food, purity of the air, over-working, and whatever tends to exalt or impair the vital energies of the people, are enumerated as so many agencies which influence these results. During fatal epidemics which alarm the public mind, Dr. E. believes that the conceptions of female children will preponderate over the males, and he adduces the births in Philadelphia and Paris, which occurred nine months after the prevalence of cholera in those cities, as an illustration of his views. The close attention which Dr. Emerson has given to the subject of vital statistics, impart great interest to these observations. Under the head of Toxicology and Miscellaneous matters, we find a brief section on the "mode of actions of poisons" by Professor James Blake, of the St. Louis University, intended to prove that the most rapidly fatal poisons act only by being absorbed into the blood—a doctrine which this gentleman endeavored to sustain by a series of experiments, an account of which was published some years ago, and which has since been controverted by Christison and Taylor, in the Edinburgh Medical and Surgical Journal. Dr. Blake has followed up the subject by more recent experiments, which have tended to confirm his original views.

The comparative effects of anæsthetics receive some attention in this department, as well as the effect of these agents on what are called sensitive plants.

A short abstract of a paper of Professor Horsford presented at the meeting of the American Association for the advancement of science, on "moisture, ammonia, and organic matter in the atmosphere," is well worthy of study, and may tend to throw some light upon the causes of epidemics and other diseases, believed to depend upon certain states of the atmosphere.

The existence of ozone in the air, and its supposed connection with cholera, are succinctly discussed by the committee, and the conclusion that "the ozone theory of cholera, on the whole, is not sustained" appears to be adopted by them.

The report closes with some eloquent remarks on the future prospects of American Medicine, and upon the vast benefits which the Association is destined to confer upon its rising fame.

The report on Practical Medicine and Epidemics from the pen of Dr. J. K. Mitchell, is chiefly occupied with an admirable history of the introduction of cholera into the United States in the year 1849, with the statistics of the various cholera hospitals in Philadelphia, the proportional mortality in the hospitals of Philadelphia, New York and Boston, the different plans of treating cholera and their results so far as statistics furnish them, the effects of certain articles in relieving certain symptoms of cholera, with a lucid exposition of the arguments in favor of the portability, and against the contagiousness of the disease.

The report is accompanied by two charts, one exhibiting the relative position on the ocean of the packet-ship New York, and the ship Swanton, when the cholera broke out upon them, on their way, the one to New York, the other to New Orleans; the other shows the date and place of occurrence of the first twenty-three cases of cholera reported to the Board of Health of Philadelphia, marked out on an outline map of the city and districts.

Some interesting facts illustrative of "the effect of special conditions upon the power of the progression of cholera," are also recorded, together with numerous examples of its non-extension from infected points and localities.

Our space will not permit more than a passing notice of this interesting paper, but we cannot refrain from a remark or two upon the question of the portability and non-contagiousness of cholera as presented by Dr. Mitchell.

While it is, we think, clearly shown by the facts collected with great care by the committee that cholera was introduced at the New York Quarantine Station, and at New Orleans, by the ships New York and Swanton, and that these vessels became infected with the disease while on the ocean 1000 miles apart, within a day of each other, we are still in the dark as to the means whereby it was introduced into Philadelphia and other infected cities, several months after its appeaeance at the localities stated.

It first occurred in Philadelphia on the 30th of May, at two opposite points of the city three and a quarter miles apart, and then appeared in different sections; for the first seventeen days no two cases occurring within a square of each other. If the disease is portable, how was it brought to Philadelphia, and why did it pursue so tortuous a course?

No evidence is furnished upon these points, except that the eleventh case which occurred on the 12th day of the epidemic visitation, had recently visited the infected city of New York.

Dr. Mitchell concludes from the facts stated, that the disease was not propagated by contagion, but that "the atmosphere of the whole city had become slightly epidemical, not being sufficiently poisonous to produce its specific effects unless aided by personal imprudence or a deleterious locality." It would seem therefore from this statement, that in the opinion of Dr. Mitchell, though cholera is portable, yet it may become epidemic without being first carried to the place where it spreads, or, in other words, that it is both portable and epidemic, but not contagious.

Now we cannot see the utility of introducing the portable theory into this discussion, in contra-distinction to the theory of contagion.

It appears to us just as easy to explain the introduction of cholera into the hospitals on Staten Island, and at New Orleans, from the two vessels arriving with cases on board, by the old idea of contagion, as by the doctrine of portability. The first cases on the landing of both of these vessels could be traced to them. One case was taken directly from the ship Swanton to the Charity Hospital at New Orleans, just as the vessel arrived; the ship New York had cases on board on her arrival at Quarantine, and the first case which appeared on shore, was in a person who had been on the ship forty-eight hours before. It must be remembered also, that the disease was confined in this latter locality to a small space, that a rigid quarantine was established between the Island and New York city, and that not a single case occurred in the city during its prevalence on the island.

We cannot see, therefore, that the introduction of the word *portable*, has any tendency to clear up the obscurity in which this subject is involved, and we are disposed to believe in the absence of farther proof, that cholera, like typhus fever, scarlet fever, and other kindred affections, is both epidemic and contagious, being greatly influenced in its progress, by those special conditions, which are known to favor the attacks of malignant disease. This view is at least as satisfactory to our mind, as that which excludes the idea of contagion, and embraces that of portability.

The committee make some brief remarks upon the occurrence of yellow fever within the past year at Charleston and Rio Janeiro. Its appearance in the latter place, being unprecedented, induced the committee to seek the aid of the Naval Bureau, in investigating this singular fact; and they have been assured of the hearty co-operation of the medical officers of the Navy in presenting the enquiry.

Scarlatina, variola, and typhoid fever, are said to have been unusually prevalent in different parts of the United States within the past year.

In tracing out the latter disease, the committee complain of the usual difficulty of deciding from the descriptions given by physicians "whether the typhoid of Louis, or a typhus state of some of the malarious fevers of the country is to be understood." With a view of rendering information more accurate upon this and other topics, they propose a distribution of subjects to special committees, to report from year to year. The committee remark, "one advantage of the distribution of subjects to special committees, would be the issue by each of an interrogative formulary, so that societies and individuals, might know how to give, the information upon which such reports could be founded. The knowledge now concealed in modest retreats, must thus be concentrated in the Association, to be, by its authority, diffused over the world."

This suggestion appears to us well worthy of the consideration of members of the Association, and will, we trust, demand special attention at an early date.

As appendices to the report, are subjoined two interesting papers one by Dr. Pancoast of Philadelphia, on the treatment of Aphonia, and the other by Dr. Reynolds of Gloucester, Massachusetts, on Epidemic Fever and Dysentery, but our limits will not permit a tice of their contents.

(To be continued.)

